# DSF Guided Refolding As A Novel Method Of Protein Production

**DOI:** 10.1038/srep18906

**Published:** 2016-01-19

**Authors:** Amadeo B. Biter, Andres H. de la Peña, Roopa Thapar, Jean Z. Lin, Kevin J. Phillips

**Affiliations:** 1 Diabetes and Metabolic Disease Program, Houston Methodist Research Institute, Houston, Texas, USA; 2 Department of Biomedical Engineering, Johns Hopkins University, Baltimore, Maryland, USA; 3 Department of BioSciences-Biochemistry and Cell Biology, Rice University, Houston, Texas, USA; 4 Department of Molecular and Cellular Biology, Baylor College of Medicine, Houston, Texas, USA

## Abstract

The Anfinsen hypothesis, the demonstration of which led to the Nobel prize in Chemistry, posits that all information required to determine a proteins’ three dimensional structure is contained within its amino acid sequence. This suggests that it should be possible, in theory, to fold any protein *in vitro*. In practice, however, protein production by refolding is challenging because suitable refolding conditions must be empirically determined for each protein and can be painstaking. Here we demonstrate, using a variety of proteins, that differential scanning fluorimetry (DSF) can be used to determine and optimize conditions that favor proper protein folding in a rapid and high-throughput fashion. The resulting method, which we deem DSF guided refolding (DGR), thus enables the production of aggregation-prone and disulfide-containing proteins by refolding from *E. coli* inclusion bodies, which would not normally be amenable to production in bacteria.

Biochemical research, as well as biomedical applications like protein-based therapeutics, depends crucially on the ability to produce proteins that are properly folded and possess native activity. Although numerous advances have greatly improved the facility of producing proteins in eukaryotic cells, protein expression in *E. coli* offers the advantage that production can be completed rapidly and at lower cost^1^,^2^. Unfortunately, most eukaryotic proteins cannot be produced recombinantly in *E. coli*. In particular, many mammalian and eukaryotic proteins require eukaryotic chaperones to fold, or contain disulfide bonds^2^,^3^ that cannot be formed in the reducing environment of the bacterial cell. Indeed, as much as 80% of recombinant protein targets will express as insoluble inclusion bodies in *E. coli*^4^. Although these inclusion bodies represent substantial pools of relatively pure protein^5^, they are misfolded, amorphous, protein aggregates^3^ that lack biological activity.

The Anfinsen hypothesis^6^ prescribes that protein folding is driven by the polypeptide assuming the most thermodynamically stable state. This dictates that all information required to fold a protein is contained within its linear amino acid sequence, and implies that it should be theoretically possible to fold all proteins *in vitro*. However, protein folding often competes with the non-productive, off-pathway processes of misfolding and aggregation^7^. Thus, *in vitro* refolding conditions must be determined that promote folding while minimizing off-pathway processes. In practice, these conditions must be determined empirically for each target protein, a process that can be time-consuming and labor-intensive^5^,^8^.

Consequently, research in this area has gone into streamlining the process of conducting and analyzing refolding attempts in a high throughput fashion^9^,^10^,^11^. In particular, refolding kits, which contain buffers and additives commonly used in refolding efforts, are now commercially available and greatly facilitate refolding trials. While these kits allow an investigator to rapidly sample refolding conditions in multi-well fashion, one must still determine which, if any, of the conditions successfully produce natively folded protein. Currently, the most advanced methods tend to use simple and nonspecific assays, such as spectroscopy^9^, solubility^12^, or elution from a nickel^13^ or hydrophobic interaction column^8^, techniques which can be readily performed in high-throughput fashion or are easily automated, as a primary screen for refolding. This primary screening is typically followed by subsequent analyses using other analytical, biological or enzymatic assays^8^,^9^,^12^,^14^,^15^. While progress has clearly been made in advancing the process of refolding, a key limitation of most reported methods is that they rely upon one or more assays that are specific to the protein of interest. This requirement limits the utility of these methods when dealing with novel proteins of interest, for which suitable assays are unlikely to exist. Further, despite recent advances, protein production by refolding is not commonly attempted and refolding is still considered a “method of last resort“^4^.

Here, we describe a novel application of differential scanning fluorimetry (DSF) screening as a method to rapidly determine and optimize refolding conditions. This approach facilitates high-throughput refolding trials in microscale, eliminating the need for target-specific assays or for subsequent low-throughput analysis. We demonstrate the generality of this approach by using it to refold various model proteins and to produce several high-value proteins that could not be expressed as soluble proteins in *E. coli*. We expect this method to greatly expand the utility of refolding as a means of protein production.

## Results

### Overview of DSF guided protein refolding (DGR)

**Figure 1:**
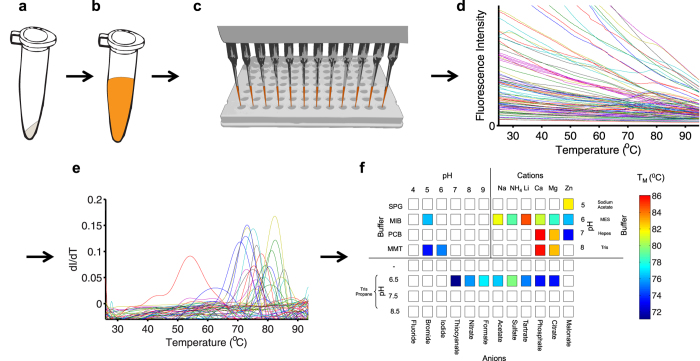
Overview of DGR. (**a**) The target protein, commonly obtained from *E.coli* inclusion bodies, is isolated. (**b**) The protein is then solubilized with a chaotropic agent such as guanidine. Sypro Orange dye is added, to facilitate the DSF assay, either at this step or following protein dilution. (**c**) Refolding is attempted by diluting the solubilized protein 20-fold into a multi-well plate containing buffers and additives, such as the PACT screen, to be tested for refolding using a DSF experiment. (**d**) The raw fluorescence melts from a 96 well DSF experiment are analyzed as described in reference (13). (**e**) The first derivative of (**d**) is calculated to determine T_M_ values. (**f**) T_M_’s are subsequently mapped to the plate to assist in identifying conditions producing apparent melting transitions. Conditions for which no unfolding transition was apparent, and thus, T_M_ values could not be calculated, are shown in white. Conditions of the 96 well PACT screen, which is the used for all subsequent refolding trials in this manuscript, are shown in (**f**). The upper left quadrant of the screen varies pH, while the upper right quadrant tests the effects of various cations at diverse pH’s, and the bottom half of the screen tests the effects of anions at 3 different pH’s and while unbuffered. Figures 1a–c were drawn by KJP.

Figure 1 presents a schematic overview of DGR. The target, which can be obtained from essentially any source, such as inactive or misfolded protein preparations, precipitates, or bacterial inclusion bodies (Fig. 1a), is first solubilized in a chaotropic agent such as urea or guanidine (Fig. 1b). Refolding is then attempted in high-throughput fashion by rapid dilution into a multi-well plate containing various buffers, salts, and additives (Fig. 1c). Addition of the dye Sypro Orange allows for individual refolding trials (wells) to be characterized via a DSF experiment using a commercial QPCR instrument, providing a diff scanning fluorescence protein unfolding or ‘melting’ trace for each condition tested. The resulting fluorescence data are then analyzed as described previously^16^, providing raw melting curves (Fig. 1d), derivatives thereof (Fig. 1e), and T_M_ values for each refolding trial (Fig. 1f).

The method readily accommodates various denaturants and conditions. Thus, in principle, any screen of buffers and additives can be used to attempt refolding. We generally initiate refolding trials using a PACT screen, which we have modified to exclude precipitants and all experiments reported in this manuscript utilize this screen. The PACT screen is a grid screen designed to systematically explore the effects of pH, anions, and cations^17^, and contains buffers and salts commonly used in protein biochemistry, refolding, and crystallization. The grid-like nature of the screen dictates that similar conditions are physically clustered in the multi-well plate; thus providing a manner of redundancy, facilitating identification of trends in the refolding data, and increasing the robustness of screening results.

While it’s relatively simple to attempt protein refolding, especially by the method of rapid dilution used here, analysis of these trials and determining which refolding attempts succeeded in producing natively folded protein, is non-trivial. DSF is a screening method that relies upon the determination of protein stability, which is measured by monitoring the temperature at which a folded protein unfolds or ‘melts’. Since this method directly monitors the folded-to-unfolded transition of proteins, we posited that it might be well suited to discriminate between trials that yielded folded protein from unsuccessful trials. Further, the method is quite tolerant to various buffers and additives that are commonly used for protein folding efforts. A common question that arises regarding DGR is, “For a novel protein for which one has no standard to compare with, how do you known what the T_M_ should be?” Since the Anfinsen hypothesis dictates that the native state of a folded protein is also the most thermodynamically stable, our assumption from the outset has been that refolding conditions that produce melts (folded-to-unfolded transitions) at the highest T_M_ values are those most likely to produce a natively folded protein product. Henceforth, we will attempt to demonstrate the general validity of this assumption, that by selecting conditions which yield cooperative melting transitions at high T_M_’s, DSF screening can be used to determine and optimize conditions for protein folding.

### DSF can readily discriminate between native, unfolded, and misfolded protein states

**Figure 2:**
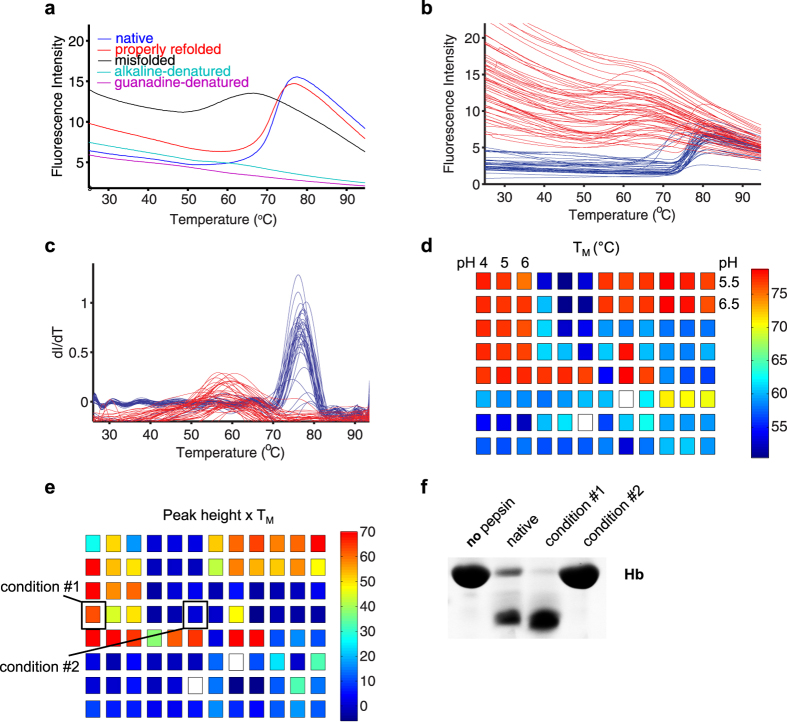
DSF can readily discriminate between folded, unfolded, and misfolded proteins. (**a**) Thermal melting profiles of natively folded, denatured, refolded, and misfolded pepsin. (**b**,**c**) Raw fluorescence traces (**b**) and the derivatives thereof (**c**) resulting from the dilution of guanidine solubilized pepsin into the 96 conditions of the PACT screen. Two distinct populations are readily apparent, corresponding to misfolded (red) and natively folded (blue) pepsin. (**d**) Mapping of T_M_ values onto the PACT screen indicates that pepsin refolds exclusively in acidic pH. (**e**) Map of T_M_ × peak height. (**f**) Protease activity towards hemoglobin (Hb), visualized by SDS-PAGE, of pepsin refolded into either condition #1 or condition #2.

As an initial test of whether DSF could be used to guide protein folding efforts, we tested the ability of DSF to discriminate between folded, unfolded, and misfolded states of the protein pepsin. Pepsin has been extensively characterized as a model protease, and is somewhat unusual in its ability to populate a stable, yet misfolded, and inactive conformation^18^. Natively folded pepsin produced an obvious and robust melting transition at 73 °C that was completely lost when denatured by exposure to pH 9.0 or the addition of guanidine (Fig. 2a). The melting transition was fully restored when pepsin was refolded from the guanidine-denatured state by dilution into a condition known to promote proper refolding (100 mM MMT buffer, pH 4.0). In contrast, misfolded pepsin, obtained by acidification of alkaline-denatured pepsin^18^,^19^, produced a less cooperative thermal melting transition at a lower T_M_ (Fig. 2a), which could be readily distinguished from that of native pepsin.

As a further test of DGR, we attempted to refold guanidine-denatured pepsin in the 96 unique conditions of the PACT screen. Analysis of the DSF data revealed that candidate refolding conditions could readily be divided into two groups: those that promoted proper refolding, resulting in high T_M_ melting traces characteristic of native pepsin, and those that yielded misfolded pepsin, resulting in broad melting transitions at lower T_M_’s (Fig. 2b,c). Mapping T_M_ values onto the PACT screen suggested that pepsin refolded exclusively in acidic conditions (Fig. 2d). Assuming peak height to be an indirect measure of refolding yield, we calculated the values of T_M_ × peak height (Fig. 2e), which suggested that highly acidic conditions (pH 4) were most effective at promoting pepsin refolding. As expected from results of the screen, preparative refolding trials of denatured pepsin into 100 MMT, pH 9.0 produced misfolded pepsin which lacked protease activity, while refolding in 100 mM MMT, pH 4.0 produced enzymatically active pepsin that efficiently degraded hemoglobin (Fig. 2f). These results demonstrate that DSF analysis can be used to discriminate between conditions that favor proper protein folding and those that do not.

### Generality of DGR

**Figure 3:**
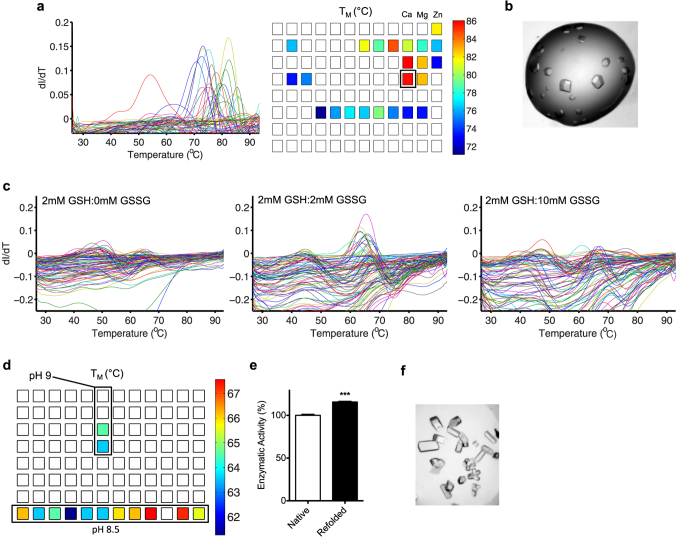
DGR of model proteins. (**a**) Results of a DGR screen of glucose isomerase. Conditions chosen for preparative refolding are noted by the black box. (**b**) Crystals of the refolded protein. (**c**) Refolding trials of denatured-reduced lysozyme under various redox conditions. (**d**) Map of T_M_’s from **c** (middle panel) suggests that denatured-reduced lysozyme refolds best at high pH in the presence of equimolar GSH and GSSG. (**e–f**) Enzymatic activity (**e**) and crystals of lysozyme (**f**) refolded from the denatured-reduced state.

In order to explore the general applicability of DGR, we attempted to determine suitable refolding conditions for several commonly used and readily available model proteins with enzymatic activity: lysozyme, xylanase, carbonic anhydrase, and glucose isomerase. These proteins either do not contain disulfide bonds, or, in the case of lysozyme, the disulfide bonds were not reduced. Refolding conditions were determined in similar fashion as outlined in Fig. 1: guanidine-denatured proteins were diluted into the PACT screen and assayed for refolding success by DSF assay. Lysozyme, xylanase, and carbonic anhydrase appeared to refold in nearly all conditions tested, as most wells had apparent melting transitions, resulting in derivatives with sharp peaks (Supplementary Fig. 1a,b). For preparative scale refolding, a refolding condition that produced a cooperative melt at a high T_M_ was selected for each enzyme, and scaled up 1000-fold. All three enzymes refolded in constitutive yield in the chosen conditions and produced a refolding product that exhibited thermal melts with T_M_ values indistinguishable from native proteins and had native enzymatic activities. Refolded lysozyme and xylanase could readily be crystallized (Supplementary Fig. 1c–e). Contrasting with the other model proteins, which appeared to refold in the majority of conditions tested, refolding trials of glucose isomerase produced apparent melting transitions in only about 20 conditions of the PACT screen, with the highest T_M_’s clustered around conditions containing divalent cations (Fig. 3a). Preparative refolding of glucose isomerase into conditions producing the highest T_M_ (100 mM Tris HCl, pH 8.0 and 200 mM CaCl_2_) yielded a protein product with the same thermal melting profile as the native enzyme (Supplementary Fig. 2b) and the refolding product was crystallizable, (Fig. 3b), suggesting the enzyme was properly refolded and homogeneous.

### Refolding disulfide-containing proteins

Secreted proteins often contain disulfide bonds and are thus unsuitable for soluble expression in *E. coli*. Further, disulfides complicate refolding efforts, as suitable redox conditions, which allow native disulfide bonds to form while minimizing the formation of improper disulfide linkages, must be determined. To test whether DGR could be used to determine conditions to refold disulfide-containing proteins, we attempted to refold lysozyme in which the four native disulfide bonds had been disrupted by reduction. As discussed previously, the refolding of lysozyme, which had been denatured but not reduced, appeared to be trivial as the enzyme seemed to refold in essentially every condition tested (Supplementary Fig. 1a). In contrast, refolding trials following reduction of the disulfide bonds in lysozyme did not appear to elicit refolding, as indicated by the complete absence of melting transitions in any condition of the PACT screen (Supplementary Fig. 3). In order to test refolding in diverse redox conditions, refolding of denatured and reduced lysozyme was again attempted in conditions of the PACT screen supplemented with various ratios of oxidized and reduced glutathione. At an equimolar ratio of oxidized and reduced glutathione, prominent melting peaks appeared (T_M_ ~ 65 °C) in alkaline conditions of the screen, suggesting that lysozyme had refolded properly in these conditions (Fig. 3c,d). Further, when the ratio of oxidized to reduced glutathione was altered to be more either more highly reducing or more oxidizing, the magnitude of the melting peaks diminished, suggesting that these conditions were sub-optimal for lysozyme refolding (Fig. 3c). Following preparative refolding in the condition producing the highest T_M_ and purification, refolded lysozyme was enzymatically active and could readily be crystallized, demonstrating that the protein was natively folded and homogenous (Fig. 3e,f). These data demonstrate that DGR can be used to determine suitable redox conditions, thus enabling the production of disulfide-bonded proteins.

### Refolding of non-model proteins from inclusion bodies

Since our experiments with model proteins suggested that DGR might be generally useful, we proceeded to test the method on several proteins that were of interest to our group, and which we were unable to produce via soluble expression in *E. coli*. In all cases, the protein was expressed in *E. coli* as insoluble inclusion bodies, purified, and subjected to DGR trials.

#### FGF19/21

Fibroblast Growth Factors 19 and 21 (FGF19 and FGF21) are small (~23 kDa) polypeptide hormones, whose metabolic effects are of interest to us and others^20^,^21^. Both growth factors contain disulfide bonds, and thus cannot be produced via soluble expression in *E. coli*. As with denatured and reduced lysozyme, variation of redox conditions was required before any conditions were discovered that produced sharp melting transitions (T_M_ ~62 °C) and suggested the production of properly refolded protein (Supplementary Fig. 4a). Preparative-scale refolding of FGF19 into a malonate containing condition that yielded amongst the highest T_M_ (Supplementary Table 1), generated refolded protein in near quantitative yield. Refolded FGF19 produced a sharp, cooperative, DSF melting transition (Supplementary Fig. 4b) and could be crystallized, suggesting the preparation to be natively folded and homogenous (Supplementary Fig. 4c). Initial DGR trials of the related growth factor, FGF21, produced only melting transitions of very low intensity (Supplementary Fig. 5a), suggestive of poor yields. Indeed, preparative scale refolding efforts resulted in heavy precipitation and a low yield (<15%) of refolded FGF21. Thus, we questioned whether it might be possible to improve refolding efficiency by further optimizing the refolding condition, a process not unlike the optimization of crystallization conditions. Hence, we selected from the PACT screen an initial ‘lead’ condition containing 200 mM sodium citrate and producing a T_M_ of ~45 °C (Supplementary Fig. 5a). By conducting refolding trials in an optimization matrix that varied pH between 8.0 and 9.0 and citrate concentrations between 0 and 800 mM, it was observed that refolding in conditions with higher pH and increased citrate concentrations resulted in DSF melts with increased T_M_’s and substantially higher magnitudes (Supplementary Fig. 5b), suggesting improved yield. Indeed, preparative refolding of FGF21 in the optimized condition (Supplementary Table 1) provided the protein in high yield (75%). These results demonstrate that DGR can be used both to determine and to optimize refolding conditions.

#### Irisin

**Figure 4:**
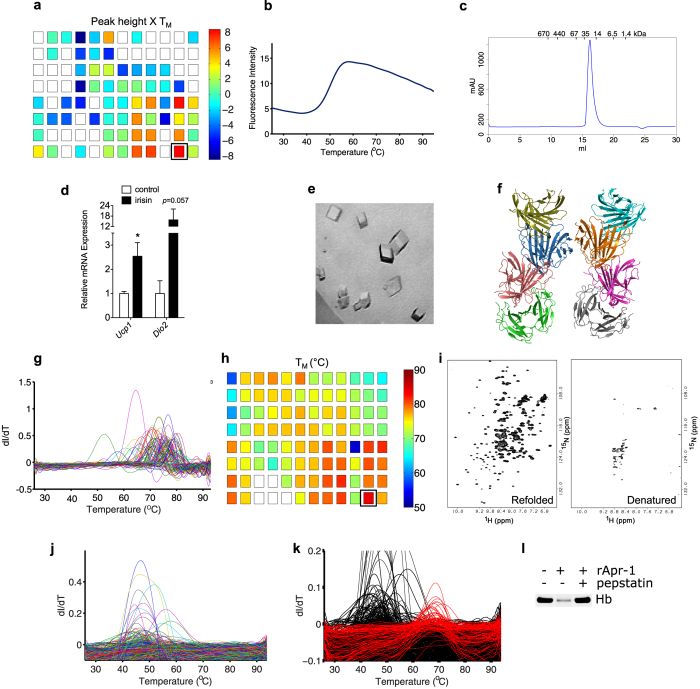
DGR of various proteins from bacterial inclusion bodies. (**a**) DGR screen of irisin; conditions chosen for preparative refolding are boxed. (**b,c**) Thermal melt (**b**) and analytical size exclusion chromatography (**c**) of refolded irisin. (**c**) Refolded irisin induced the expression of known target genes in cell culture. (**e,f**) Crystals (**e**) and molecular replacement solution (**f**) of refolded irisin. (**g–i**) DGR (**g,h**) and HSQC spectra of refolded ^15^N-SYLF (**i**). (**j**) Overlay of refolding trials of the catalytic domain of Apr-1, representing ~1,000 unique conditions. (**k**) Refolding trials of a propeptide-containing Apr-1 construct (red) superimposed upon the data from (**j,** black) demonstrate that the propeptide containing construct refolds to produce melts at a substantially higher T_M_. (**l**) Refolded propeptide containing Apr-1 is catalytically active based on its ability to proteolyze hemoglobin (Hb) and is inhibited by the specific inhibitor pepstatin, as visualized by SDS-PAGE.

Irisin is a small polypeptide hormone, reported to mediate some of the metabolic benefits of exercise^22^, which we had previously found to express as predominantly insoluble in *E. coli*. The results of DGR trials of irisin are shown in Fig. 4. A condition containing 100 mM bis-Tris propane HCl, pH 8.5 and 200 mM sodium citrate was chosen for preparative-scale refolding, as it produced the largest combination of T_M_ and peak height (Fig. 4a) This condition produced refolded irisin in over 95% yield. Refolded irisin produced a cooperative DSF melt with a T_M_ of 50 °C (Fig. 4b), and appeared homogeneous via size-exclusion chromatography, with an apparent molecular weight of 28 kDa, suggesting the protein to be dimeric (Fig. 4c). Refolded irisin was biologically active, as it was capable of inducing known target genes, Ucp1 and Dio2, in cell culture. (Fig. 4d). We were able to crystallize refolded irisin in the space group *P*2_1_, with unit-cell parameters *a* = 93.90 Å; *b* = 132.94 Å; *c* = 110.20 Å and α = 90°; β = 97.7°; γ = 90° (Fig. 4e). In the course of our experiments, the crystal structure of irisin was published^23^, allowing us to finalize structure determination by molecular replacement, revealing 16 copies, or eight dimers, in the asymmetric unit (Fig. 4f).

### SYLF

The SYLF domain is a 21-kDa domain of unknown function, found in such human proteins as SH3YL1. SYLF expressed as completely insoluble when produced in *E. coli.* Results of DGR of SYLF are shown in Fig. 4. SYLF appeared to refold in numerous conditions (Fig. 4g), with the most stable products being formed in conditions containing buffered 200 mM sodium citrate (Fig. 4h). Thus, preparative refolding was conducted in 100 mM bis-Tris propane HCl, pH 8.5 and 200 mM sodium citrate. While SYLF appeared to refold constitutively in this condition, and was homogenous on SEC, the protein did not crystallize despite extensive crystallization trials. As the SYLF domain is of unknown function, functional studies could not be used to demonstrate that the protein was natively folded. To ensure that the protein was properly folded, and to demonstrate the potential of DGR as a means to produce isotopically labeled proteins for NMR, ^15^N-SYLF was produced by refolding from *E. coli* inclusion bodies. The HSQC resonance spectra of refolded ^15^N-SYLF were sharp and well-resolved in the amide proton region from 6.6–10.1 ppm (Fig. 4i), demonstrating that refolded SYLF is structured. More importantly, this result demonstrates that DGR may be used to obtain isotopically labeled proteins from inclusion bodies.

#### Apr-1

Finally, we attempted to produce the protease Apr-1 by DGR. Apr-1 is a 50 kDa aspartic protease being developed as a potential vaccine against hookworm (*Necatur americanus*) infection^24^. Like other aspartic proteases, Apr-1 is synthesized as an inactive zymogen with an N-terminal propeptide that is cleaved off to yield the mature enzyme. Notably, the catalytic domain of Apr-1 has been challenging to produce via traditional expression methods^24^, perhaps because the propeptide may be necessary for proper folding and maturation is likely complicated by the presence of four disulfide bonds, as indicated by homology modeling. Refolding of the mature catalytic domain of Apr-1 (74-417) was initially attempted in the PACT screen supplemented with various ratios of redox agents, including oxidized and reduced glutathione, TCEP, and copper chloride. Additionally, refolding was attempted in numerous commercially available crystallography screens, also supplemented with reducing and oxidizing agents, altogether representing over one thousand individual refolding trials. In a small number of these trials, melting transitions were observed between 40 °C and 60 °C (Fig. 4j). However, in preparative refolding efforts, none of these conditions yielded enzymatically active Apr-1. In comparison, refolding trials of a larger, propeptide containing Apr-1 construct into the 96 conditions of the PACT revealed several conditions with melting transitions at significantly higher T_M_’s between 63 °C and 71 °C (Fig. 4k). Preparative refolding of this construct into 100 mM Tris-HCl, pH 8.0 and 200 mM magnesium chloride yielded active protease with a T_M_ of 68 °C, whose activity is inhibited by pepstatin, a specific inhibitor of aspartic proteases (Fig. 4j). These results suggest that Apr-1 may require the propeptide for proper folding, and demonstrate that DGR can be used to discriminate between various protein expression constructs for refolding.

## Discussion

We have demonstrated the utility of DSF as a tool to rapidly determine and optimize refolding conditions for many different proteins, including those that are aggregation-prone or disulfide-bonded. Upon scale up, refolding products were suitable for various biochemical and biophysical applications, including crystallography, NMR, and activity and cell-based assays. One particular application, which we demonstrate by producing ^15^N-labeled SYLF (Fig. 4i), where we forsee that DGR might have a significant impact, is in the production of isotopically labeled proteins for NMR. In general, proteins destined for NMR analysis, must be uniformly isotopically labeled. However, uniform labeling in insect- and mammalian cell cultures is highly challenging, because, unlike *E. coli*, these cultures cannot commonly be grown in deuterium oxide^25^, or isotope-labeled minimal media^25^. Labeling media for insect and mammalian cells have recently become available commercially; however these media are generally quite expensive^25^,^26^,^27^ and suffer from problems of ill-defined composition^25^,^26^ and variable labeling efficiency^26^,^27^,^28^. Hence, the refolding of isotopically labeled protein from inclusion bodies represents an appealing and inexpensive alternative means to obtain uniformly labeled proteins for NMR studies.

We emphasize that for none of the proteins produced from inclusion bodies in this work did we have active protein ‘standards’ by which to assist in the analysis of the DSF guided screening of refolding conditions. Instead, refolding conditions were chosen solely based on the intrinsic traits of the fluorescence traces, specifically T_M_, magnitude, and cooperativity of the unfolding melts. It should be noted that the T_M_ values that result from FGR are in no way a measure of the folding process itself, but are instead a measure of the stability of the protein solutions that results from the refolding attempts. Since the diverse pH and buffer conditions used for refolding can significantly affect protein stability^16^, the resulting T_M_ value is a convolution of two factors: a) did the protein refold and b) what is the stability of the refolded species in a given well or condition? What this means in practice, is that for most proteins the actual T_M_ values are not so important as identifying those conditions that do indeed produce T_M_’s. That is, finding conditions that yield melting (folded-to-unfolded) transitions for which T_M_ values can be assigned and, moreover, determining those that produce melting transitions of high magnitude, indicative of a large amount of folded protein, or higher yield. What prevents us from suggesting that optimization should be based solely on magnitude is that disulfide containing proteins can refold into non-native states that tend to exhibit melting transitions of low cooperativity and low T_M_ values, which are, thus, readily avoided by selecting conditions with higher T_M_’s.

Thus, we suggest that one should optimize for refolding by finding conditions that produce highly cooperative melts at high T_M_’s and high magnitudes. We believe that our results here demonstrate practically that conditions suitable for protein refolding can indeed by chosen solely by selecting conditions that maximize these parameters: T_M_, magnitude, and cooperativity and thus, do not require any a priori knowledge of the target protein. Thus, a key advantage of using DSF as an analytical method to monitor protein refolding is that it is target-independent, requiring only that the target protein produce an observable thermal melting transition. This obviates the need to develop and optimize target-specific assays for each protein of interest. With the exception of high detergent concentrations, DSF is generally tolerant of most buffers and additives commonly used for protein folding. Further, DSF can much more readily be adapted to various high-throughput and robotic platforms than other methods that have been used to assess refolding success^29^.

We were surprised to find that the inability to express a protein solubly in *E. coli* does not necessarily suggest that the protein construct will be challenging to refold *in vitro*. In particular, both irisin and SYLF expressed almost exclusively as insoluble in *E. coli*, perhaps suggesting that their folding of these proteins may be difficult or require eukaryotic chaperones for efficient refolding. However, the two proteins appeared quite amenable to refolding efforts, as both were found to refold in nearly all conditions of the PACT screen and could be refolded in essentially constitutive yield. Thus, given the short time and little expense required to attempt DGR, it may be well advised to routinely attempt refolding trials for those proteins that express as insoluble in *E. coli.*

As the ability to rapidly initiate protein production is a competitive advantage to both academic and commercial labs, a key benefit of the method presented here is the speed and economy with which new proteins can be produced. Once insoluble protein is in hand, which can be obtained rapidly via inclusion bodies, DGR trials can readily be performed and analyzed in a period of hours. Thus, we expect that DGR could greatly expand the utility of using protein refolding to produce recombinant proteins, particularly by allowing disulfide containing and aggregation prone proteins to be produced in *E. coli*, for which production would generally be relegated to eukaryotic expression systems.

## Methods

### Preparation of denatured enzymes and inclusion bodies

Denatured model enzymes were obtained by dissolving lysozyme, xylanase, glucose isomerase, and carbonic anhydrase in denaturing buffer (100 mM Tris HCl, pH 7.4; 6 M guanidine HCl) to a final concentration of 6 mg/mL. Denatured reduced lysozyme was obtained by dissolving lysozyme in denaturing buffer supplemented with 40 mM glutathione. Pepsin was dissolved in 100 mM MIB buffer, pH 4.0 and 6 M guanidine HCl.

To obtain inclusion bodies, bacterial expression plasmids containing human irisin (FNDC5 residues 32–143), FGF19 (40–175), FGF21 (42–177), and *N. americanus* Apr-1 (30–417 or 74–417) were transformed into *E. coli* BL21 (DE3). Transformants were grown to OD_600_ ~ 1.0 in LB media supplemented with appropriate antibiotics, and induced for 3–4 hours with 0.2 mM IPTG. Cells were harvested by centrifugation, resuspended in lysis buffer (40 mM Tris HCl, pH 7.4; 400 mM NaCl; 6 mM β-Mercaptoethanol) and passed 3–4 × through a Microfluidizer at 20,000 psi. Inclusion bodies were harvested by centrifugation at 4,000 × g, washed 4 × in lysis buffer, and solubilized in denaturing buffer. Solubilized inclusion bodies were purified by standard Ni-affinity chromatography under denaturing conditions. Finally, purified inclusion bodies were buffer-exchanged into denaturing buffer. ^15^N-labeled SYLF (residues 33–231 of a hypothetical protein from *C. candidatus thermophilus*) was obtained in the same manner except that cultures were grown in minimal media containing ^15^NH_4_Cl.

### DGR

Guanidine-denatured enzymes or purified inclusion bodies were refolded in 96-well plates by diluting 2 μL of protein into 40 μL of the PACT screen with or without reducing or oxidizing agents. Plates were left at room temperature for two hours, and then centrifuged at 3,700 × g for 20 minutes to pellet any precipitate that may have formed. DSF assays were then performed and analyzed as described previously^16^ using a mixture of 20 μL of refolding product and 2 μL of 25 × Sypro orange. For clarity, spurious traces were omitted from plots of raw DSF and derivative data. Preparative refolding conditions (Supplementary Table 1) were selected from the initial screens by choosing conditions that produced the highest T_M_ values with cooperative melts of high magnitude and scaled up as appropriate to obtain amounts sufficient for crystallization trials, NMR and activity or cell-based assays. If necessary, refolded proteins were concentrated by Ni-affinity chromatography and then buffer-exchanged into 20 mM TrisHCl, pH 7.4, 40 mM NaCl and 6 mM β-mercaptoethanol.

### Enzymatic assays

Lysozyme activity was determined by clearing of a 3 mg/mL suspension of *M. lysodeikticus* cells^30^, as measured by decrease in absorbance at 450 nm. Carbonic anhydrase activity was determined by measuring the release of nitrophenol from *p*-nitrophenyl acetate^31^, as measured by increase in absorbance at 348 nm. Glucose isomerase activity was measured in a coupled reaction, in which the isomerization of xylose to xylulose was detected by the reduction of xylulose by sorbitol dehydrogenase, using NADH as donor^32^. The disappearance of NADH was measured by decrease in absorbance at 340 nm. Xylanase activity was measured by clearing of a 5 mg/mL suspension of xylan^33^, as measured by decrease in absorbance at 600 nm. Pepsin and Apr-1 protease activity was observed by degradation of hemoglobin, as visualized on SDS-PAGE^24^,^34^.

### NMR Spectroscopy

Sensitivity-enhanced (^1^H, ^15^N)-HSQC spectra were acquired at 30 °C on a Varian Inova 600 MHz spectrometer equipped with a 5 mm cryogenic ^1^H{^13^C/^15^N} probe on uniformly ^15^N-labeled SYLF at concentrations of 0.9 mM – 1.1 mM. Refolded ^15^N-SYLF was kept in 40 mM potassium phosphate buffer, pH 6.5, 50 mM NaCl, and 10% D_2_O, while denatured ^15^N-SYLF was kept in 6M guanidine HCl. NMR data were apodized using a square-shifted sine bell (72–90°) and analyzed using Felix (Felix NMR).

## Additional Information

**How to cite this article**: Biter, A. B. *et al.* DSF Guided Refolding As A Novel Method Of Protein Production. *Sci. Rep.* **6**, 18906; doi: 10.1038/srep18906 (2016).

## Supplementary Material

Supplementary Information
