# Supplementary material for: DSF Guided Refolding As A Novel Method Of Protein Production
Source: Sci Rep. 2016 Jan 19;6:18906. doi: 10.1038/srep18906 (PMC4726114; doi:10.1038/srep18906)
Supplement: Supplementary Information [file srep18906-s1.pdf]

# DSF Guided Refolding As A Novel Method Of Protein Production

Amadeo B. Biter, Andres H. de la Peña, Roopa Thapar, Jean Z. Lin, Kevin J. Phillips

Supplementary Information

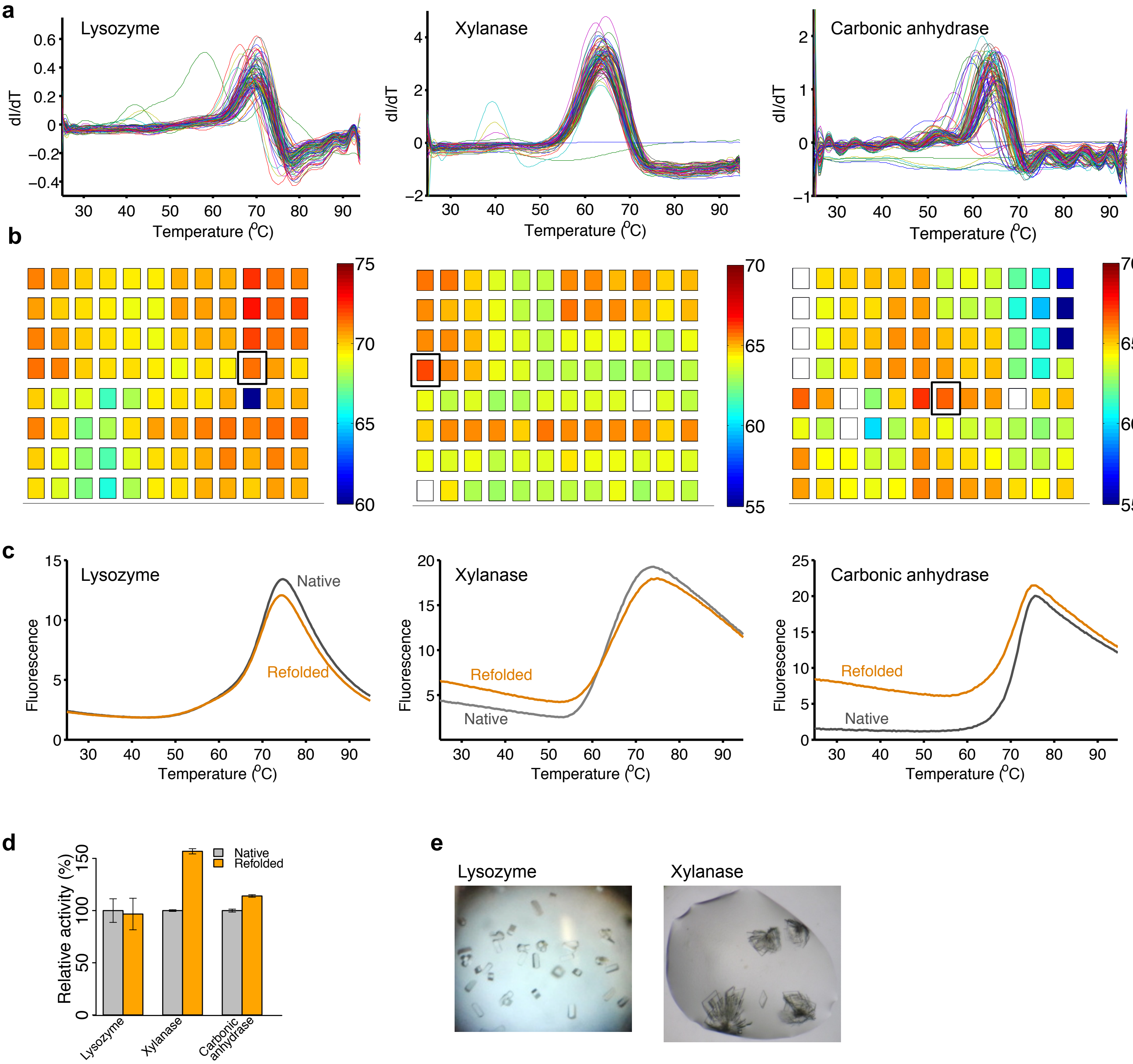


**Supplementary Fig. 1. DGR of model enzymes.** (**a,b**) DSF (derivative) traces (**a**) and heat maps (**b**) of of refolding trials of non-reduced lysozyme, xylanase, and carbonic anhydrase in conditions of the PACT screen. Conditions chosen for preparative scale refolding are boxed. (**c**) Thermal melt traces of ezymes produced by preparative refolding. (**d**) Crystals of refolded lysozyme and xylanase.

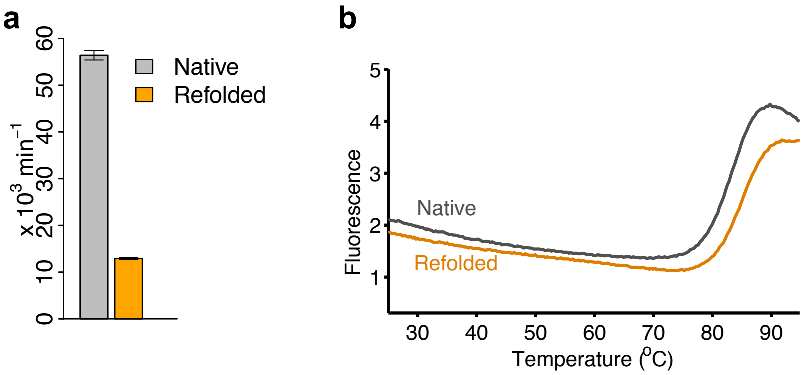


**Supplementary Fig. 2. Refolded glucose isomerase.** (**a-b**) Enzymatic activity (**a**) and DSF melt (**b**) of refolded and native glucose isomerase. Calcium, which is present in the refolding buffer, is a known inhibitor of glucose isomerase activity.

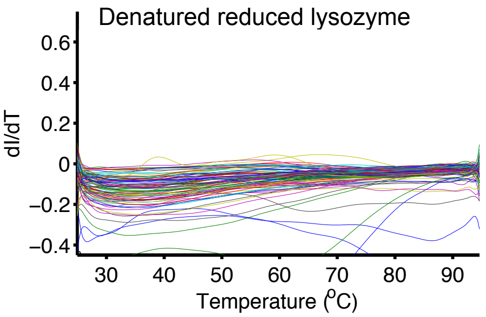


**Supplementary Fig. 3. Effect of disulfide reduction on lysozyme refolding.** DSF (derivative) traces indicate that denatured-reduced lysozyme does not refold in conditions of the PACT screen in the the absence of redox agents.

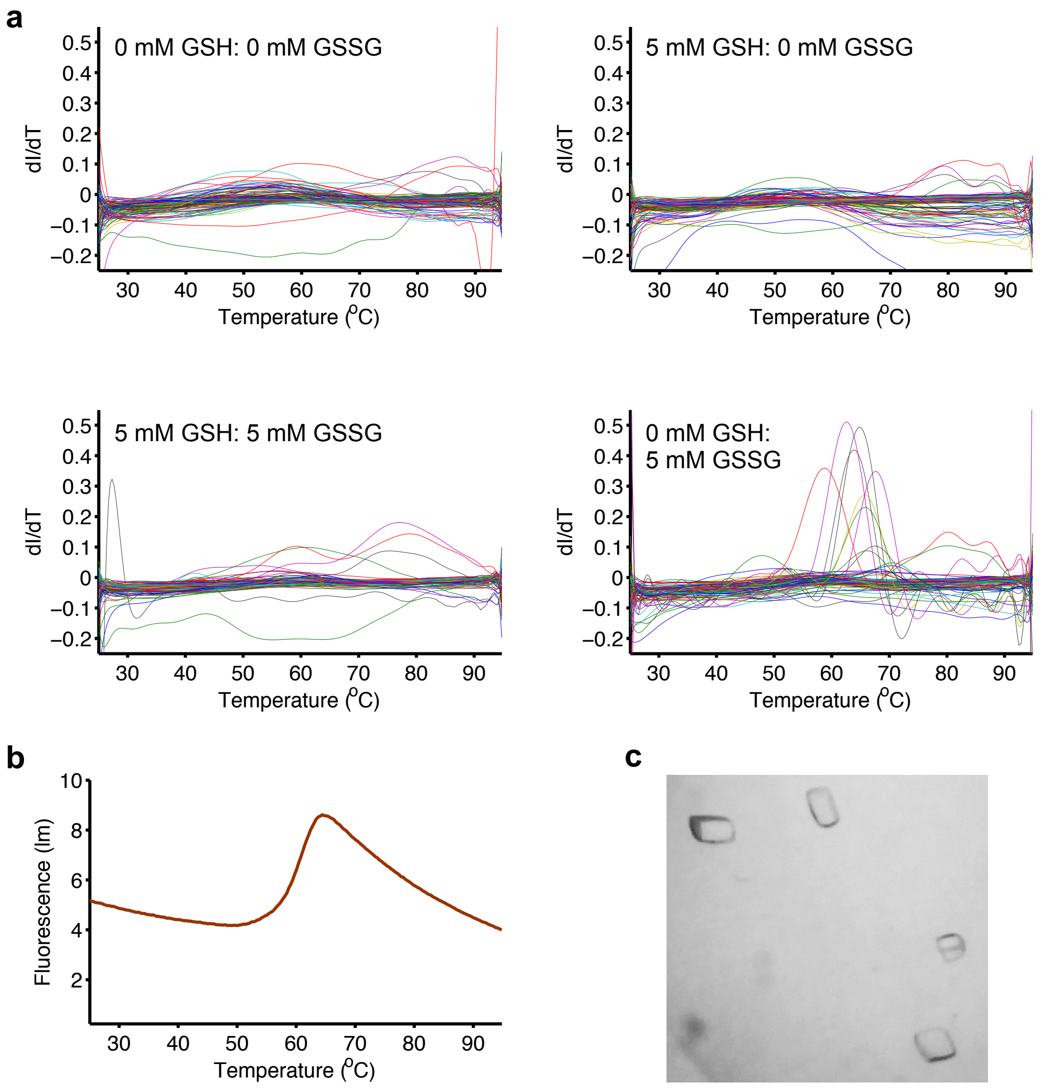


**Supplementary Fig. 4. DGR of FGF19.** (**a**) DSF (derivative) traces reveal that FGF19 refolds only when the PACT screen is supplemented with 5 mM GSSG and no GSH (bottom right). (**b**) DSF melt of refolded FGF19. (**c**) Crystals of refolded FGF19.

Supplementary Table 1. Preparative refolding conditions, as selected or optimized from analytical DGR screens.

| Target protein | Refolding condition |
|---|---|
| Lysozyme | 100 mM Tris HCl, pH 8.0<br>200 mM magnesium chloride |
| Reduced lysozyme | 200 mM sodium potassium tartrate<br>2 mM GSH<br>2 mM GSSG |
| Xylanase | 100 mM MMT, pH 4.0 |
| Glucose isomerase | 100 mM Tris HCl, pH 8.0<br>200 mM calcium chloride |
| Carbonic anhydrase | 200 mM sodium acetate |
| Pepsin | 100 mM MMT, pH 4.0 |
| FGF19 (40-175) | 100 mM bis-Tris propane HCl, pH 8.5<br>200 mM sodium malonate<br>2.5 mM GSSG |
| FGF21 (42-177) | 100 mM bis-Tris propane HCl, pH 9.0<br>400 mM sodium citrate<br>0.2 mM GSH<br>0.2 mM GGSG |
| Irisin (FNDC5 32-143) | 100 mM bis-Tris propane HCl, pH 8.5<br>200 mM sodium citrate |
| $^{15}$N-SYLF (33-231) | 200 mM sodium citrate |
| Apr-1 (30-417) | 100 mM Tris-HCl, pH 8.0<br>200 mM magnesium chloride |

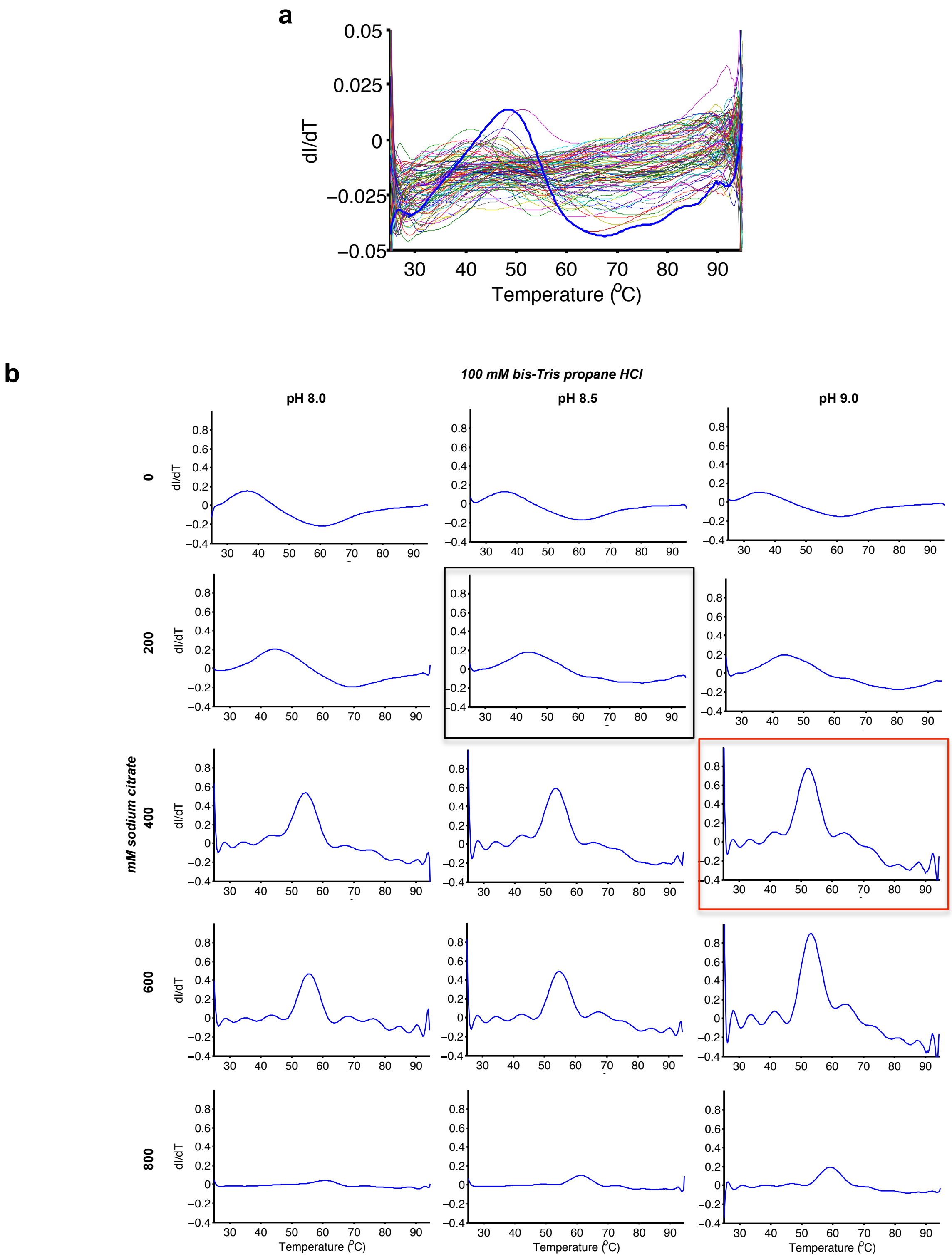


**Supplementary Fig. 5. DGR based optimization of FGF21 refolding conditions.** (**a**) DSF (derivative) traces from FGF21 refolding trials in the PACT screen supplemented with 0.2 mM GSH and 0.2 mM GSSG. The initial 'lead' condition chosen for optimization is shown in bold blue. (**b**) Optimization of conditions around the original refolding condition (boxed in black) identifies conditions with DSF melts of higher magnitude. The condition chosen for preparative scale refolding is denoted by a red box.
